# Intranasal mRNA-LNP vaccination protects hamsters from SARS-CoV-2 infection

**DOI:** 10.1126/sciadv.adh1655

**Published:** 2023-09-22

**Authors:** Gabriela Baldeon Vaca, Michelle Meyer, Ana Cadete, Chiaowen Joyce Hsiao, Anne Golding, Albert Jeon, Eric Jacquinet, Emily Azcue, Chenxia Monica Guan, Xavier Sanchez-Felix, Colette A. Pietzsch, Chad E. Mire, Matthew A. Hyde, Margaret E. Comeaux, Julie M. Williams, Jean C. Sung, Andrea Carfi, Darin K. Edwards, Alexander Bukreyev, Kapil Bahl

**Affiliations:** ^1^ Moderna Inc., Cambridge, MA, USA.; ^2^ Department of Pathology, University of Texas Medical Branch, Galveston, TX, USA.; ^3^ Galveston National Laboratory, Galveston, TX, USA.; ^4^ Department of Microbiology and Immunology, University of Texas Medical Branch, Galveston, TX, USA.

## Abstract

Intranasal vaccination represents a promising approach for preventing disease caused by respiratory pathogens by eliciting a mucosal immune response in the respiratory tract that may act as an early barrier to infection and transmission. This study investigated immunogenicity and protective efficacy of intranasally administered messenger RNA (mRNA)–lipid nanoparticle (LNP) encapsulated vaccines against severe acute respiratory syndrome coronavirus 2 (SARS-CoV-2) in Syrian golden hamsters. Intranasal mRNA-LNP vaccination systemically induced spike-specific binding [immunoglobulin G (IgG) and IgA] and neutralizing antibodies. Intranasally vaccinated hamsters also had decreased viral loads in the respiratory tract, reduced lung pathology, and prevented weight loss after SARS-CoV-2 challenge. Together, this study demonstrates successful immunogenicity and protection against respiratory viral infection by an intranasally administered mRNA-LNP vaccine.

## Teaser

SARS-CoV-2 intranasal mRNA-LNP vaccine is immunogenic and protects against respiratory viral infection in an animal model.

## INTRODUCTION

Disease caused by respiratory pathogens remains a pre-eminent threat to global public health (*1*). With more than 600 million cases and 6.5 million deaths reported worldwide as of November 2022, the ongoing coronavirus disease 2019 (COVID-19) pandemic caused by severe acute respiratory syndrome coronavirus 2 (SARS-CoV-2) is the most current and vivid example of the impact of respiratory diseases on global populations (*2*). Before the COVID-19 pandemic, upper and lower respiratory tract infections were responsible for more than 17.7 billion cases and 2.5 million deaths globally and primarily caused by viruses and bacteria such as *Streptococcus pneumoniae*, respiratory syncytial virus, and influenza virus (*3*). There remains a continual risk of emerging respiratory infectious diseases (*4*), as evidenced by evolving SARS-CoV-2 variants in the current COVID-19 pandemic and by notable prior pandemics caused by pathogens such as influenza virus (*2*). Vaccination remains a pivotal strategy to address infectious disease–related morbidity and mortality (*5*), with a need for innovative vaccination strategies and technologies that can be deployed quickly and establish robust local mucosal immune responses in the upper respiratory tract to impede infection and transmission (*6*).

Now, most licensed vaccines against respiratory diseases are administered intramuscularly, which primarily induce systemic immunity while also eliciting some immunity at the mucosal sites targeted by respiratory pathogens (*6*–*9*). Intranasal vaccination can induce both systemic and local mucosal immune responses and is a promising approach to combat respiratory pathogens as it has the potential to limit infection and minimize transmission by establishing early, local immunity at key infection sites (*7*–*15*). This approach could also increase vaccination rates and compliance with recommended schedules, as its minimally invasive delivery may facilitate administration without the need for trained health care personnel (*7*, *16*, *17*). In addition, intranasal vaccination via a device that creates a spray or aerosol could potentially bypass injection-associated phobias that are responsible for vaccine hesitancy (*18*). While few intranasal vaccines are now authorized (*6*, *9*), the continued emergence of SARS-CoV-2 variants shifted attention to vaccination strategies that may better limit transmission and slow variant progression. Consequently, multiple intranasal SARS-CoV-2 vaccines based on viral vector, live attenuated, or protein subunit designs are currently in preclinical and clinical development (*7*), with two mucosal SARS-CoV-2 viral-vectored vaccines having recently received regulatory approval in China and India (*19*).

The mRNA–lipid nanoparticle (LNP) encapsulated vaccines have already demonstrated the ability to protect against infectious respiratory pathogens, as shown by currently available COVID-19 vaccines: mRNA-1273 [Spikevax; Moderna, Inc., Cambridge, MA, USA (*20*)) and BNT162b2 (Comirnaty; Pfizer Inc., New York, NY, USA; BioNTech Manufacturing GmbH, Mainz, Germany] (*21*–*26*). Moreover, mRNA-LNP vaccines do not induce a vector-specific immune response and thus have high potential for repeat administration without diminishment of effect caused by antivector immunity (*27*–*29*). Further benefits include that mRNA is also noninfectious and nonintegrative (*29*), while LNPs can be modified for delivery of mRNA to specific cells, tissues, and organs (*30*, *31*). In this report, we demonstrate that a two-dose regimen of intranasally administered mRNA-based SARS-CoV-2 vaccines is immunogenic and protects against viral infection in a Syrian golden hamster model.

## RESULTS

### Intranasal mRNA-LNP vaccination induces binding and neutralizing antibody responses in sera

**Fig. 1.**
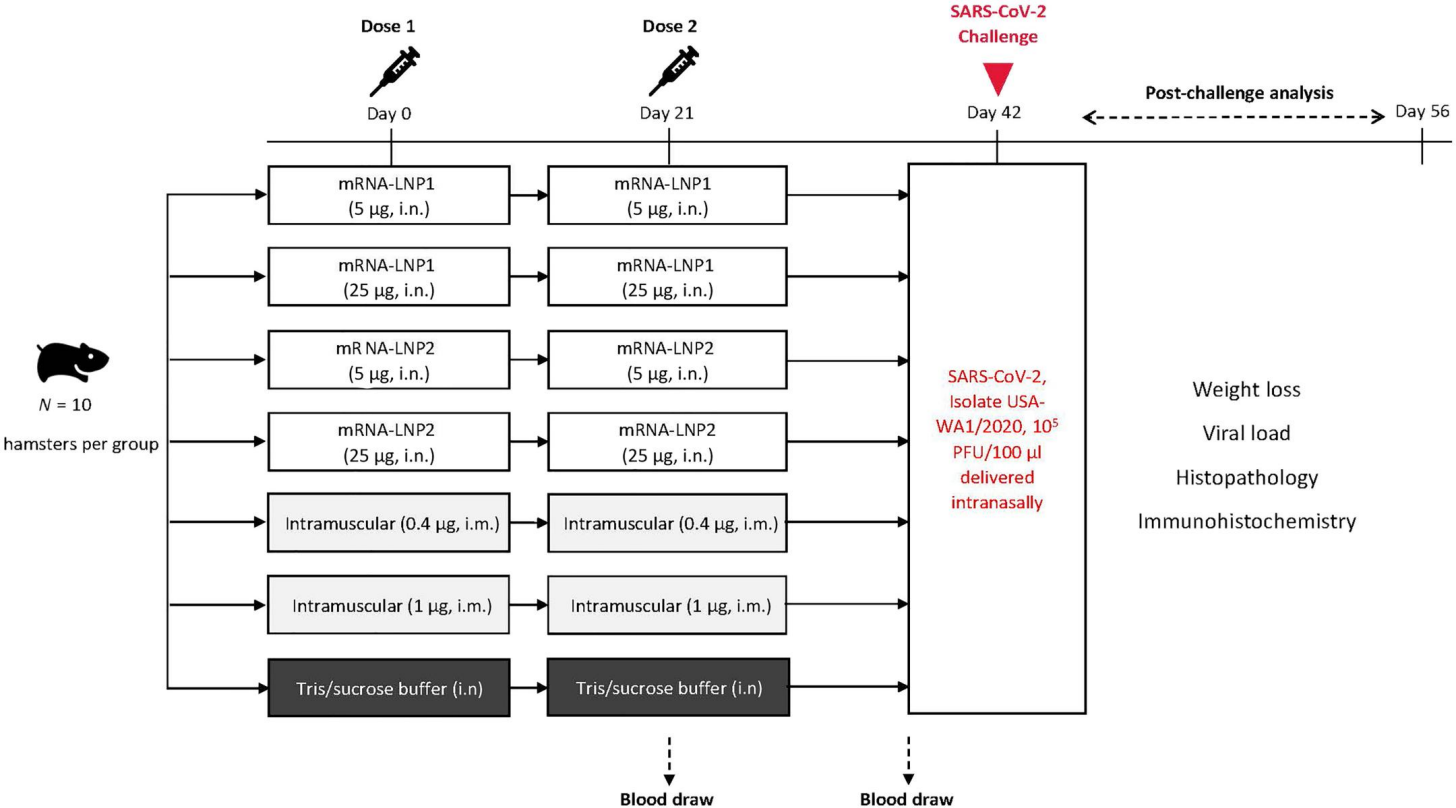
Study design. Intranasal vaccination of an mRNA-based SARS-CoV-2 vaccine was evaluated in Syrian golden hamsters. Hamsters (*n* = 10 per group) were intranasally immunized with two doses (days 0 and 21) of vaccines (5 or 25 μg) formulated in two different LNP compositions or were mock-vaccinated with two doses of tris/sucrose buffer administered intranasally; separate groups of animals were intramuscularly immunized with two doses of vaccine (0.4 or 1 μg). Sera were collected approximately 3 weeks after dose 1 (before dose 2 on day 21) and approximately 3 weeks after dose 2 (day 41). At day 42, hamsters were intranasally challenged with SARS-CoV-2 (2019-nCOV/USA-WA1/2020). Post-viral challenge assessments included viral load and histopathology [3 days (day 45) and 14 days (day 56) after challenge], immunohistochemistry (3 and 14 days after challenge), and body weight (daily after challenge). i.m, intramuscular; i.n, intranasal; PFU, plaque-forming units.

To assess the immunogenic potential of intranasally administered N1-methyl-pseudouridine–modified mRNA-LNPs, we developed SARS-CoV-2 vaccines formulated with two different LNP compositions: mRNA-LNP1 and mRNA-LNP2. mRNA-LNP1 is similar in composition to the LNP used in mRNA-1273, with similar but chemically distinct ionizable lipids, and mRNA-LNP2 is a composition further developed for improved respiratory tract delivery through the addition of a cationic lipid. All vaccines encoded a prefusion-stabilized SARS-CoV-2 spike (S) protein, stabilized with six proline mutations (*32*). Syrian golden hamsters (*n* = 10 per group) were vaccinated 3 weeks apart with two doses of either mRNA-LNP vaccines (5 or 25 μg) or with tris/sucrose buffer (mock-vaccinated) via the intranasal route (days 0 and 21; Fig. 1). As a control, two groups of hamsters were intramuscularly vaccinated with 0.4 μg (suboptimal dose) or 1 μg (protective dose) of vaccine with the same mRNA included in the intranasal compositions but formulated with the preclinical version of the same LNP used in injectable mRNA-1273. Immunogenicity was assessed at approximately 3 weeks after dose 1 (day 21) and approximately 3 weeks after dose 2 (day 41); S-specific serum immunoglobulin G (IgG)– or IgA-binding antibody responses (ancestral) were measured by enzyme-linked immunosorbent assay (ELISA), and serum neutralizing antibody titers (ancestral and omicron B.1.1.529) were measured by a plaque reduction neutralization test.

**Fig. 2.**
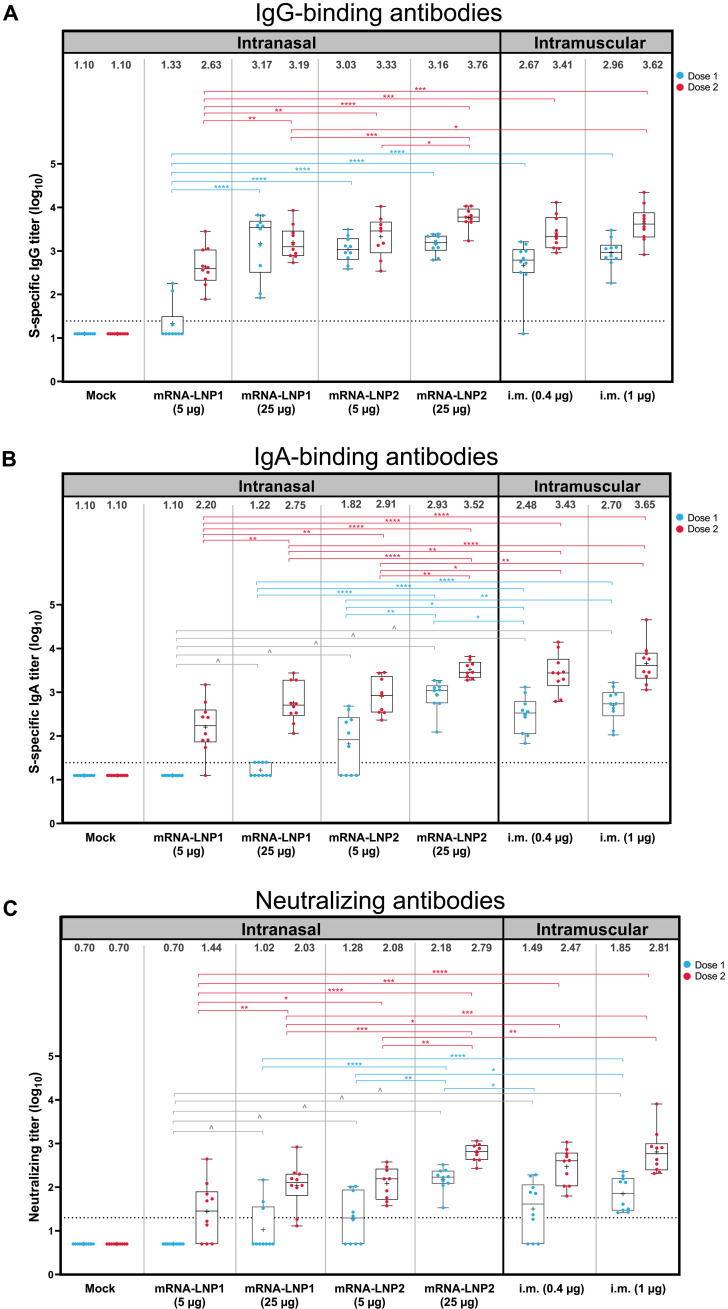
Serum immune responses against ancestral SARS-CoV-2 after intranasal vaccination. (**A**) S-specific serum binding IgG, (**B**) S-specific serum binding IgA, and (**C**) serum neutralizing antibody reciprocal end-point titers (log scale) against ancestral SARS-CoV-2 at 3 weeks after dose 1 (day 21) or 3 weeks after dose 2 (day 41) are shown by vaccine group. Animal-level data are shown as dots (*n* = 9 to 10 animals per group), with boxes and horizontal bars denoting the interquartile range (IQR) and median, respectively, and whiskers representing the maximum and minimum values. Geometric mean titers for each vaccine group are indicated by the plus (+) symbol of each boxplot, with the exact values shown above each vaccine group. Horizontal dotted lines represent the lower limit of detection (LLOD). Bayesian linear mixed model was used to model IgG, IgA, and neutralization titers. Holm’s method was used to adjust *P* values for multiple comparisons. *P < 0.05, **P < 0.01, ***P < 0.001, and ****P < 0.0001. Results of statistical comparisons between groups are shown in tables S1 to S3. ^^^ denotes antibodies that were under the limit of detection for all hamsters in the mRNA-LNP1 (5 μg) group after dose 1, which had a much lower antibody level compared to other groups. S2-P, S-protein with two proline mutations.

Three weeks after the first dose, both intranasal vaccines (25-μg dose level) elicited high S-specific serum IgG-binding titers comparable to intramuscular controls (0.4 and 1 μg). At the 5-μg dose level, mRNA-LNP2 induced similar titers to mRNA-LNP2 (25 μg) and to intramuscular controls (0.4 and 1 μg); mRNA-LNP2 titers at the lower 5-μg dose were significantly higher than mRNA-LNP1 titers (adjusted *P* < 0.0001; Fig. 2A and table S1). After the second dose, S-specific IgG titers generally increased across all vaccine groups and dose levels, with mRNA-LNP2 eliciting significantly higher titers than mRNA-LNP1 at the corresponding dose levels (5 μg, adjusted *P* < 0.01; 25 μg, adjusted *P* < 0.001).

A single intranasal administration of mRNA-LNP2 (5 and 25 μg) elicited S-specific serum IgA-binding antibody titers in sera (Fig. 2B), with the 25-μg dose level eliciting higher (adjusted *P* < 0.05) or similar titers as the intramuscular controls (0.4 and 1 μg, respectively; table S2). mRNA-LNP1 at the 5- and 25-μg dose levels elicited lower titers than intramuscular controls and respective mRNA-LNP2 doses. A second dose of either intranasal vaccine composition increased IgA-binding titers, with the 25-μg dose levels eliciting significantly higher titers than the respective 5-μg dose level (adjusted *P* < 0.01). In addition, mRNA-LNP2 (25 μg) elicited higher or comparable IgA titers to intramuscular control groups (0.4 and 1 μg) after either dose.

In addition to S-specific binding titers, neutralizing antibody responses in sera were evaluated (Fig. 2C). Three weeks after the first dose, titers were not detected in some hamsters after mRNA-LNP1 (25-μg dose), mRNA-LNP2 (5-μg dose), or in the lower dose level group of the intramuscular controls (0.4 μg). No hamsters administered mRNA-LNP1 (5 μg) had detectable titers after the first dose. However, all hamsters vaccinated with mRNA-LNP2 (25 μg) had neutralizing antibody titers that were significantly higher (*P* < 0.05) than other intranasally vaccinated groups and were similar to titers of the intramuscular controls (0.4 and 1 μg, respectively; table S3). Neutralizing antibody titers increased for all vaccine groups after the second dose. mRNA-LNP2 (5 and 25 μg) induced significantly higher titers than mRNA-LNP1 at the respective dose level (5 μg, adjusted *P* < 0.05; 25 μg, adjusted *P* < 0.001). Two doses of mRNA-LNP2 (25 μg) induced similar neutralizing titers to intramuscular controls (0.4 and 1 μg).

**Fig. 3.**
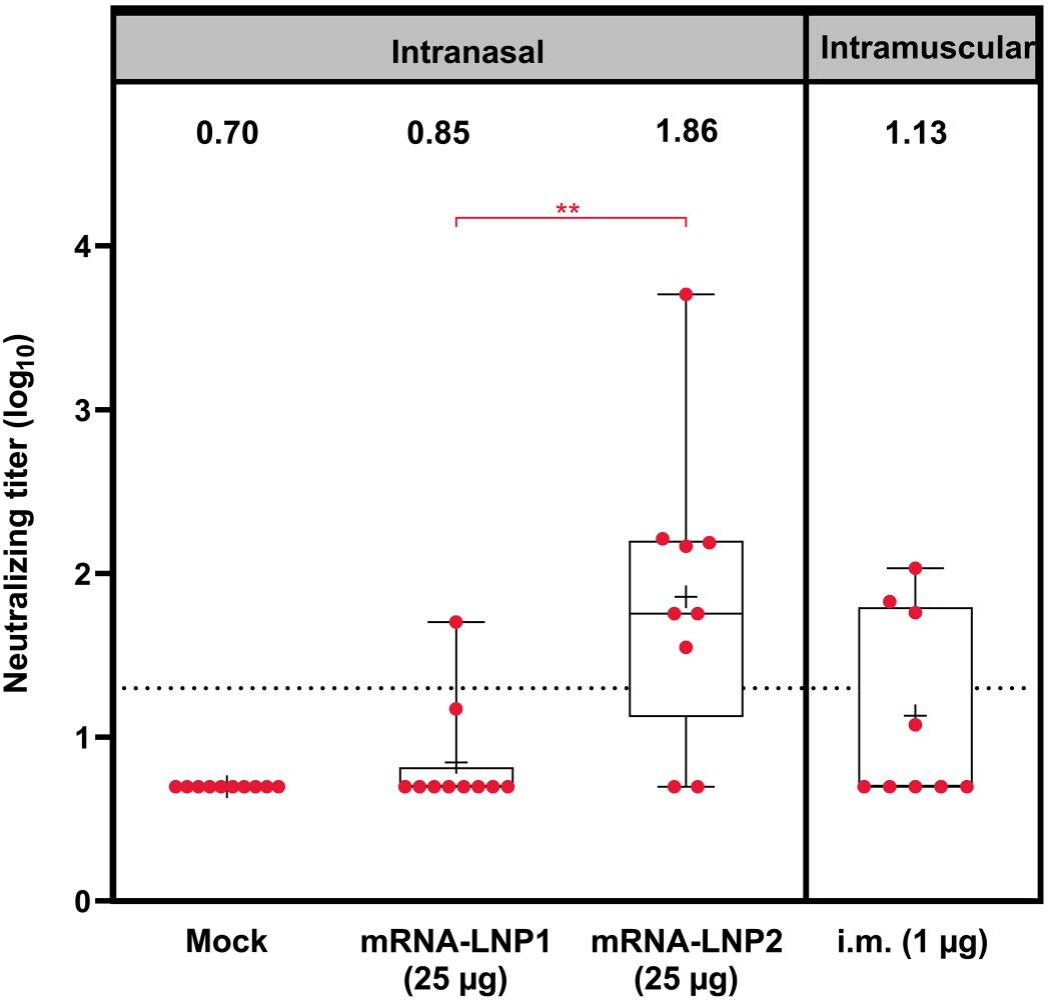
Serum neutralizing antibody titers against SARS-CoV-2 omicron B.1.1.529 after intranasal vaccination. Serum neutralizing antibody titers (log scale) against SARS-CoV-2 omicron B.1.1.529 at 3 weeks after dose two are shown by vaccine group. Animal-level data are shown as dots (*n* = 9 to 10 animals per group), with boxes and horizontal bars denoting the IQR and median, respectively, and whiskers representing the maximum and minimum values. Mean titers for each vaccine group are indicated by the plus (+) symbol, and geometric mean titers are stated above each boxplot. Horizontal dotted lines represent the LLOD. Bayesian linear mixed model was used to model neutralization titers. Holm’s method was used to adjust *P* values for multiple comparisons. **P < 0.01.

To assess for cross-neutralizing responses against emergent SARS-CoV-2 variants, neutralizing antibody titers against the omicron variant (B.1.1.529) were assessed in serum samples collected 3 weeks after the second dose of mRNA-LNP1 (25 μg), mRNA-LNP2 (25 μg), and intramuscular control vaccination (1 μg). Hamsters vaccinated with mRNA-LNP2 (25 μg) had significantly greater levels of omicron-specific neutralizing antibodies compared to hamsters vaccinated with mRNA-LNP1 (25 μg) (adjusted *P* < 0.01) (Fig. 3).

### Intranasal mRNA-LNP vaccination limits viral replication in the respiratory tract and protects against disease

Three weeks after the second dose (day 42), all vaccinated and mock-vaccinated hamsters were challenged intranasally with 10^5^ plaque-forming units (PFU) of SARS-CoV-2 (isolate USA-WA1/2020; Fig. 1). This isolate was selected for challenge as ancestral SARS-CoV-2 isolates are more pathogenic and drive more severe disease in hamsters than omicron lineage viruses (*33*, *34*). Viral loads in nasal turbinates and lung were then assessed 3 days (day 45; *n* = 5 animals per group) and 14 days after challenge (day 56; *n* = 5 animals per group), and the body weight was evaluated daily.

**Fig. 4.**
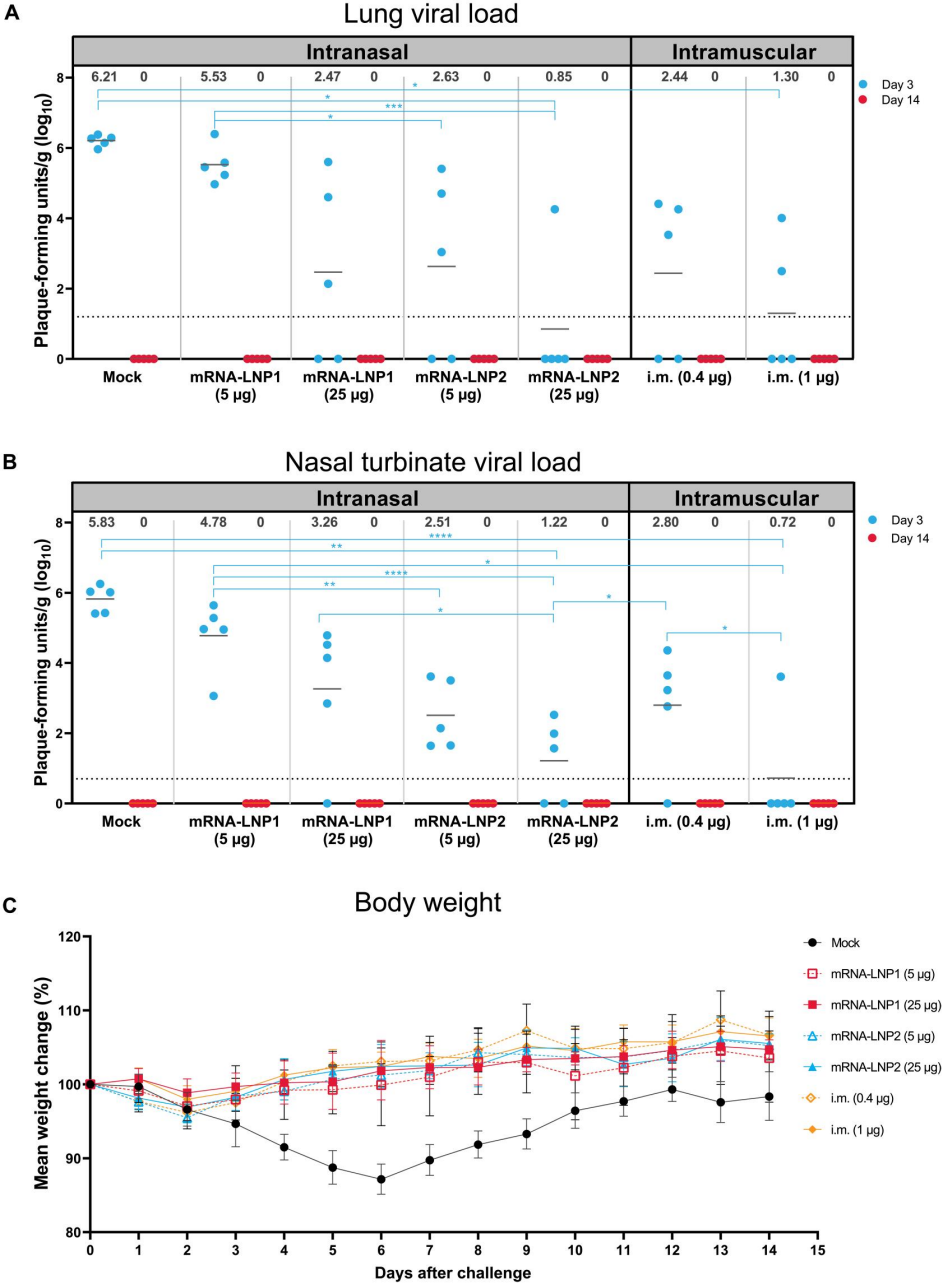
Viral load and weight loss characteristics after SARS-CoV-2 challenge in vaccinated hamsters. (**A**) Viral load (PFU per gram of tissue) in the lungs and (**B**) viral load in the nasal turbinates of mock-vaccinated and vaccinated hamsters at 3 and 14 days after SARS-CoV-2 challenge. Animal-level data are shown as dots (*n* = 5 animals per group), with gray lines representing the geometric mean titer for each group; exact values are shown above each vaccine group. Ordinary linear regression and statistical comparisons were only performed for viral loads at day 3 after challenge, as viral loads at day 14 were zero for all hamsters. Šidák’s method was used to adjust *P* values for multiple comparisons. *P < 0.05, **P < 0.01, ***P < 0.001, and ****P < 0.0001. Results of statistical comparisons between groups are shown in tables S4 to S5. (**C**) Mean percentage of weight change (error bars represent SEM) over 14 days after SARS-CoV-2 challenge in mock-vaccinated and vaccinated hamsters. SEM, standard error of the mean.

At 3 days after SARS-CoV-2 challenge, intranasally vaccinated hamsters had lower viral loads in both the lung and nasal turbinates relative to mock vaccination, as determined by plaque assay (Fig. 4, A and B, respectively). In the lung, viral loads were below the levels of detection in four of five hamsters vaccinated with mRNA-LNP2 (25 μg), which was significantly reduced relative to mock-vaccinated controls (*P* < 0.05; table S4). Viral loads were not detected in two of five hamsters vaccinated with mRNA-LNP2 (5 μg) or mRNA-LNP1 (25 μg), similar to the suboptimal dose in the intramuscular controls (0.4 μg, two of five animals). At the 1-μg intramuscular dose level, which is considered protective in this hamster model (*35*), viral load in the lungs was significantly reduced compared with mock-vaccinated controls (*P* < 0.05), with three of the five vaccinated hamsters having no detectable virus. Overall, viral loads in the lung were lower among hamsters intranasally administered mRNA-LNP2 than mRNA-LNP1 at the respective dose levels, which was significant at the 5-μg dose level (*P* < 0.05). Similarly, in nasal turbinates, viral loads were undetected in one of five hamsters vaccinated with mRNA-LNP1 (25 μg) and two of five hamsters vaccinated with mRNA-LNP2 (25 μg); loads were significantly lower with mRNA-LNP2 (25 μg) than mock vaccination (*P* < 0.01; table S5). Viral titers among hamsters intranasally vaccinated with the 5 μg-dose level of either intranasal composition remained detectable at 3 days after infection, but titers were numerically lower relative to mock vaccination and were generally similar to the lower dose intramuscular control (0.4 μg). Overall, viral reduction in the lungs and nasal turbinates of intranasally vaccinated groups was comparable to intramuscularly vaccinated control groups. By 14 days after challenge, SARS-CoV-2 virus was not detectable in the lung or nasal turbinates of any intranasally or intramuscularly vaccinated hamsters, including mock-vaccinated animals (Fig. 4, A and B).

Viral load in respiratory tissues was also determined through assessment of viral subgenomic RNA (sgRNA) levels by quantitative reverse transcription polymerase chain reaction (qRT-PCR). Corroborating the plaque assay results, all vaccinated hamsters at 3 days after SARS-CoV-2 challenge had slightly lower viral sgRNA levels relative to mock-vaccinated controls, regardless of dosage and route of administration (fig. S1). By 14 days after challenge, sgRNA was not detectable in the lung or nasal turbinates of any hamsters, including those mock vaccinated.

SARS-CoV-2 infection was performed with a dose known to result in disease characteristics such as weight loss in Syrian golden hamsters (*36*). Over the course of infection, mock-vaccinated hamsters experienced a maximum mean (± SE) weight loss of 12.9% (±1.02) by day 6 after challenge (Fig. 4C). Comparatively, all intranasally or intramuscularly vaccinated hamsters maintained their body weights over the 14-day post-challenge period.

### Intranasal mRNA-LNP vaccination reduces severity of viral pathology in the lungs

In the Syrian golden hamster model, SARS-CoV-2 infection with ancestral strains causes severe pathological lesions in the lung tissue by 3 days after infection that typically begins to resolve by 10 days after infection (*36*). Therefore, to examine the ability of intranasal mRNA-LNP vaccination to reduce lung pathology after infection, histopathological examination of the lower left lobe of the lung of hamsters was performed at 3 and 14 days after challenge.

**Fig. 5.**
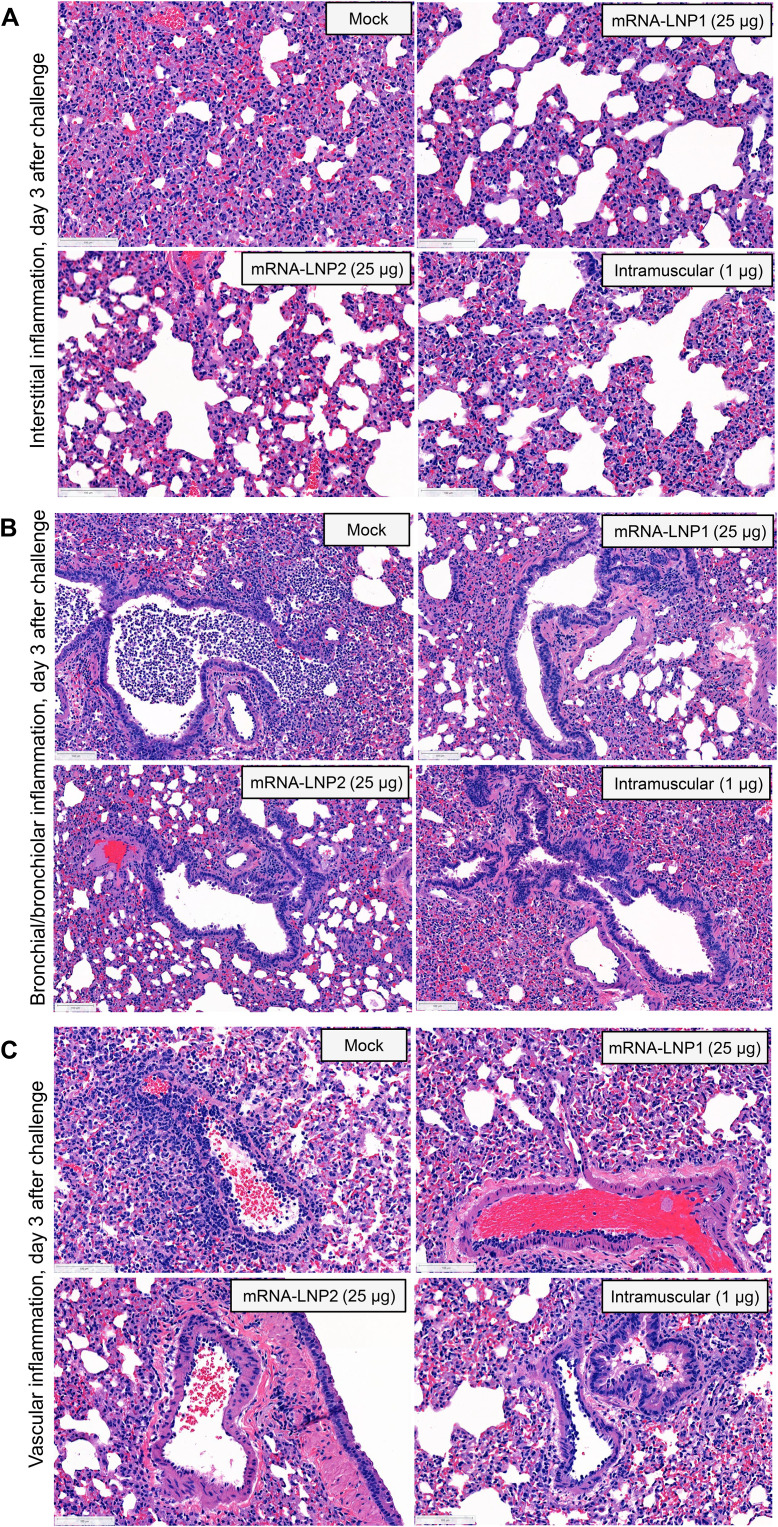
Pulmonary histopathological characteristics at 3 days after SARS-CoV-2 challenge in vaccinated hamsters. Lung sections from hamsters at 3 days after SARS-CoV-2 challenge were stained with hematoxylin and eosin. Representative images are shown for mock-vaccinated, intranasally vaccinated (25 μg), or intramuscularly immunized (1 μg) hamsters. (**A**) Pulmonary parenchyma show moderate, interstitial infiltration by mixed inflammatory cells within alveolar walls, multifocal deposits of fibrin, and alveolar hemorrhage. (**B**) Airways including bronchi and bronchioles were frequently obstructed by high numbers of neutrophils in mock-vaccinated hamsters. Note the lack of this suppurative inflammation in vaccinated hamsters. (**C**) Vascular and perivascular mixed cell infiltrates were observed in medium to large-sized blood vessels. Note the decreased severity of vascular inflammation in vaccinated hamsters. Scale bars, 100 μm.

Three days after infection, all vaccinated and mock-vaccinated hamsters exhibited acute pulmonary parenchymal tissue damage and inflammation. There were regionally extensive areas of interstitial infiltration by mixed inflammatory cells, alveolar accumulation of fibrin, hemorrhage, infiltration of bronchial/bronchiolar epithelium by neutrophils, large clusters of intraluminal neutrophils within bronchi/bronchioles with or without epithelial degeneration/necrosis, and vascular inflammation. However, there were vaccine group- and dose-dependent differences in severity. Hamsters vaccinated with high dose levels of either intranasal (25 μg) or control intramuscular (1 μg) vaccine compositions had similar levels of pulmonary parenchymal inflammation (Fig. 5A) to mock-vaccinated controls but exhibited lower severity scores for bronchial/bronchiolar inflammation (Fig. 5B) and vascular inflammation (Fig. 5C). No major differences in lung histopathology were observed for the lower dose levels compared to mock vaccination (table S6). Major histopathological findings and severity scores for each group and dose level at 3 days after challenge are summarized in table S6.

Fourteen days after SARS-CoV-2 infection, there were still regionally extensive areas of interstitial inflammation for all hamsters regardless of administration route or dose level (table S6). However, fibrin accumulation, hemorrhage, bronchial/bronchiolar inflammation, and vascular inflammation regressed, with evidence of tissue recovery such as type II pneumocyte hyperplasia. Nonetheless, compared with mock vaccination, all vaccinated groups exhibited lower severity of pulmonary inflammation irrespective of vaccine group or dose level (table S6). Histopathology at 14 days after challenge for high dose levels are shown in fig. S2.

**Fig. 6.**
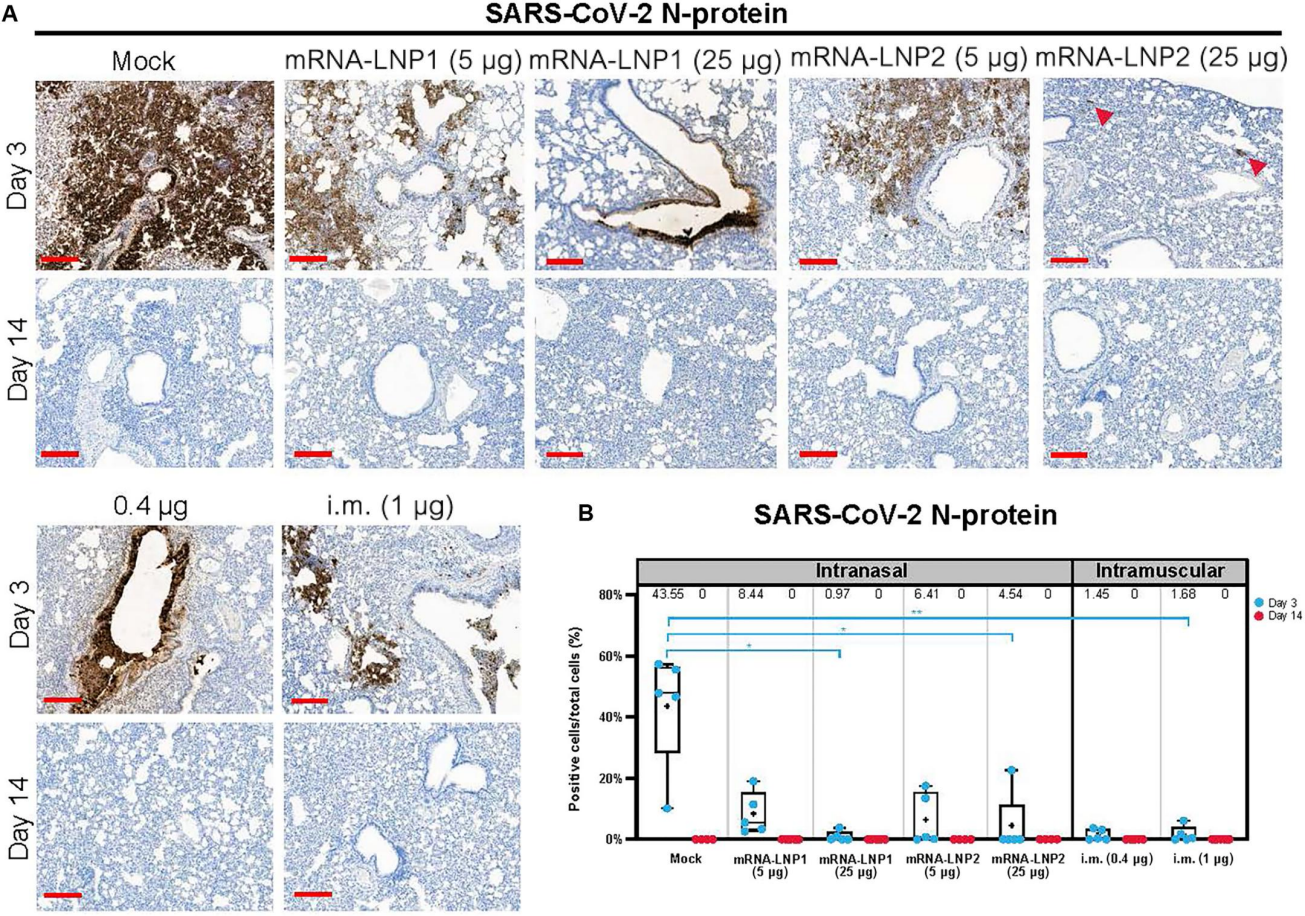
Immunohistochemistry for SARS-CoV-2 N-protein in lung after SARS-CoV-2 challenge. Lung sections from hamsters necropsied at 3 and 14 days after SARS-CoV-2 challenge were stained with an antibody raised against the SARS-CoV-2 N-protein. (**A**) Representational images of lungs from mock-vaccinated, intranasally vaccinated [mRNA-LNP1 or mRNA-LNP2 (5 and 25 μg)], or intramuscularly vaccinated (0.4 and 1 μg) hamsters. Arrowheads designate areas of positive signal within tissue. (**B**) Quantification of N-protein^+^ cells by vaccine group. Animal-level data are shown as dots (*n* = 4 to 5 animals per group), with boxes and horizontal bars denoting the IQR and median, respectively, and whiskers representing the maximum and minimum values. Mean values are provided above each plot. Scale bars, 200 μm. *N* = 5 animals per group. Kruskal-Wallis nonparametric test was implemented for statistical analysis to accommodate for small sample sizes per group and subsequent small percentage of N-protein^+^ cells within vaccinated animals. *P < 0.05 and **P < 0.01. N protein, nucleocapsid protein.

In addition, lung tissue samples were stained for the SARS-CoV-2 nucleocapsid protein (N-protein) by immunohistochemistry to identify cells infected with SARS-CoV-2 (Fig. 6A). Three days after challenge, all five mock-vaccinated hamsters had N-protein^+^ cells (group mean ± SEM: 43.5 ± 8.6% positive cells of total cells quantified; Fig. 6B and table S7). While N-protein was detected in the lung tissue of some vaccinated hamsters, vaccinated groups had a lower percentage of N-protein^+^ cells compared to mock-vaccinated controls (mRNA-LNP1: 5 μg, 8.4 ± 3.1%; 25 μg, 1.0 ± 0.7%; mRNA-LNP2: 5 μg, 6.4 ± 3.8; 25 μg, 4.5 ± 4.5%; intramuscular: 0.4 μg, 1.5 ± 0.8; 1.0 μg, 1.7 ± 1.2). The reduced percentage of N-protein^+^ cells relative to mock-vaccinated controls was significant for higher dose level groups, regardless of administration route [mRNA-LNP1 (25 μg), *P* = 0.017; mRNA-LNP2 (25 μg), *P* = 0.033; intramuscular (1 μg), *P* = 0.004]. Percentage of N-protein^+^ cells for lower dose level groups was not significantly different from mock-vaccinated controls. Notably, the mRNA-LNP2 (25 μg) group had four of the five hamsters with <1% of N-protein^+^ cells, with one hamster having 22.6% N-protein^+^ cells. Relative to mock vaccination, both low and high dose levels of intranasal vaccines reduced the percentage of N-protein^+^ cells in the lung, while the higher dose levels better ameliorated the pathologic manifestation. By 14 days after challenge, no groups were positive for N-protein, indicating virus clearance from the lungs (Fig. 6, A and B).

Lung tissue samples from hamsters vaccinated with higher dose levels of intranasal vaccines (25-μg groups) were also stained at 3 days after viral challenge for ionized calcium-binding adapter molecule 1 (IBA1) by immunohistochemistry to detect macrophage infiltration (percentage of positive cells) and activation (staining intensity). Compared to mock vaccination control, mRNA-LNP1 (25 μg) and intramuscular vaccinated hamsters (1 μg) had significantly decreased mean (± SD) percentage of IBA1^+^ cells [mock: 18.99 ± 4.58%; mRNA-LNP1 (25 μg): 7.10 ± 1.60% (adjusted *P* = 0.0037); mRNA-LNP2 (25 μg): 9.41 ± 2.75%; and intramuscular (1 μg): 8.63 ± 3.27% (adjusted *P* = 0.0333)] (fig. S3). In addition, IBA1 staining intensity was significantly decreased in mRNA-LNP1 (25 μg) and intramuscular vaccinated hamsters (1 μg) compared to mock vaccination control [*H* scores (mean ± SD), mock: 34.51 ± 12.73; mRNA-LNP1 (25 μg): 10.74 ± 2.85 (adjusted *P* = 0.0060); mRNA-LNP2 (25 μg): 14.72 ± 4.32; and intramuscular (1 μg): 13.57 ± 6.05 (adjusted *P* = 0.0431)].

### Intranasal mRNA-LNP induces local B cells in the lungs

**Fig. 7.**
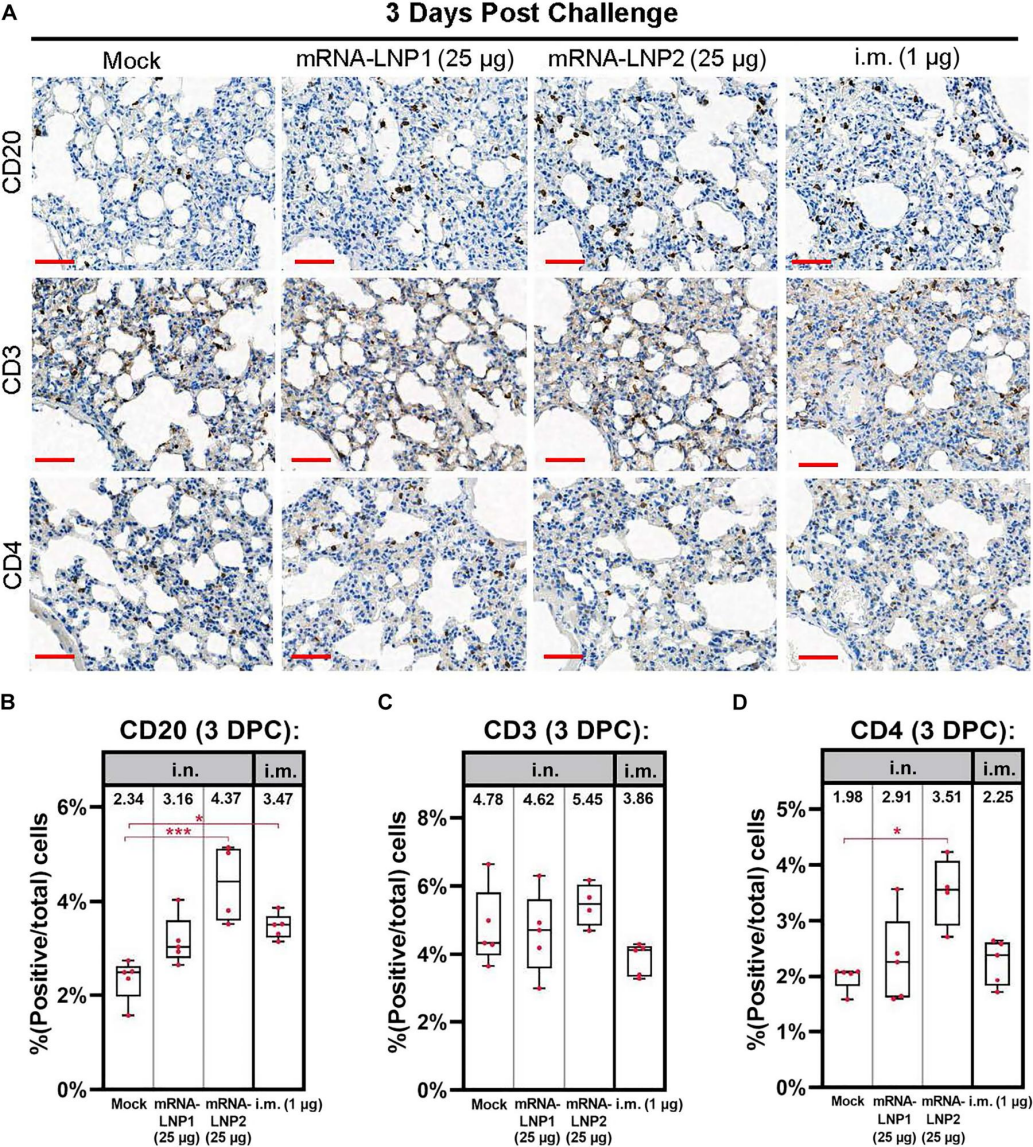
Immunohistochemistry of lymphocyte immune cells at 3 days after SARS-CoV-2 challenge. Lungs sections from hamsters necropsied at 3 days after SARS-CoV-2 challenge were stained for B cell marker CD20, general T cell marker CD3, and helper T cell marker CD4. (**A**) Representational images of hamster lung with two doses of tris/sucrose, mRNA-LNP1 (25 μg), mRNA-LNP2 (25 μg), or intramuscular composition (1 μg). Quantification of cells positive for (**B**) CD20, (**C**) CD3, and (**D**) CD4 in each of the above represented groups. Scale bars, 50 μm. Animal-level data are shown as dots (*n* = 4 to 5 animals per group), with boxes and horizontal bars denoting the IQR and median, respectively, and whiskers representing the maximum and minimum values. Means are stated above each boxplot. Kruskal-Wallis nonparametric test was implemented for statistical analysis to accommodate for small sample sizes per group. *P < 0.05, **P < 0.01, and ***P < 0.001. DPC, days post challenge.

Lung tissue samples collected at 3 days after SARS-CoV-2 challenge were also stained for markers of lymphocytes. A significantly increased percentage of CD20^+^ B cells were detected in the lung tissue of mRNA-LNP2 (25 μg) and intramuscular vaccinated hamsters (1 μg) relative to mock-vaccinated controls [mock: 2.34 ± 0.45%; mRNA-LNP1 (25 μg): 3.16 ± 0.52%; mRNA-LNP2 (25 μg): 4.37 ± 0.83% (adjusted *P* = 0.0007); and intramuscular (1 μg): 3.47 ± 0.27% (adjusted *P* = 0.0146)] (Fig. 7). There were no significant differences between vaccine groups for a general T cell marker (CD3), including for the intramuscular control group (1 μg) (Fig. 7). The percentage of CD4^+^ helper T cells was significantly increased in hamsters vaccinated with mRNA-LNP2 (25 μg) relative to mock-vaccinated controls [mock: 1.98 ± 0.23%; mRNA-LNP1 (25 μg): 2.29 ± 0.80%; mRNA-LNP2 (25 μg): 3.51 ± 0.62% (adjusted *P* = 0.0456); and intramuscular (1 μg): 2.25 ± 0.41%] (Fig. 7).

## DISCUSSION

Intranasal vaccine regimens may establish local immunity at upper respiratory sites and act as an early, protective barrier to reduce viral infection and subsequent transmission (*7*, *8*, *10*, *11*). However, vaccine development for intranasal administration is challenging. The respiratory tract is protected by a slightly acidic mucosal layer containing proteolytic enzymes that form a barrier over the epithelial cell lining that undergoes continuous mucosal clearance (*8*). These mechanisms act to defend against entry of respiratory pathogens but can subsequently prohibit antigen delivery during intranasal vaccination (*8*). Thus, novel technologies are needed to overcome these physiological barriers to advance intranasal vaccination and protect against respiratory disease.

Multiple intranasal vaccines against SARS-CoV-2 (using adenoviral vectors, live attenuated recombinant viruses, or adjuvant protein subunits) are under investigation in both preclinical and clinical studies (*7*). Further, two viral-vectored mucosal vaccines were recently approved as booster doses in India (iNCOVACC, intranasal delivery through nasal drops; Bharat Biotech International Limited, Hyderabad, India) (*37*) and China (Convidecia Air, nebulized vaccine for inhalation through the mouth; CanSino Biologics Inc., Tianjin, People’s Republic of China) (*38*). Collectively, preclinical studies examining intranasal SARS-CoV-2 vaccines have demonstrated induction of both systemic and local antibody responses and subsequent protection against SARS-CoV-2 infection (*10*, *15*, *39*–*41*), providing overall support for this vaccination route. However, findings from a recent phase 1 clinical trial on an adenovirus-vectored COVID-19 vaccine (ChAdOx1 nCoV-19) highlight the difficulties of translating animal research to humans, as the intranasal vaccine did not consistently elicit robust mucosal or systemic immune responses in vaccine naive or previously vaccinated participants (*42*) despite promising preclinical data (*15*).

An mRNA-LNP–based approach for intranasal vaccination to respiratory pathogens, including SARS-CoV-2, may offer additional advantages over more traditional vaccine development platforms (*7*, *17*). For example, mRNA-encoded antigens more closely resemble the structure and presentation of viral proteins expressed during a natural infection (*43*). In addition, an mRNA-based approach uses a single vaccine platform across different pathogens (*43*), with this platform enabling flexible antigen design, inclusion of multiple or modified antigens, and rapid incorporation of sequence substitutions that may be needed due to the emergence of variants (*43*). mRNA-based vaccines may also potentially minimize safety concerns associated with more traditional approaches used for mucosal vaccines, including those reliant on a live attenuated virus that have a theoretical risk of reverting to its pathogenic form. In addition, mRNA vaccines have a vector-less approach and thus can avoid the potential for diminished immunogenicity with repeat dosing sometimes observed with vector-based vaccines. Moreover, the utility of intramuscularly administered mRNA vaccines against respiratory pathogens such as SARS-CoV-2 has been established, demonstrating robust immune responses and high real-world effectiveness against disease (*21*, *22*, *44*). However, adapting this platform for intranasal vaccination still poses known technical challenges, including identifying the key target cells in the respiratory tract as well as sufficient mucoadhesion and penetration to access these cells. Additional unknown hurdles to developing an immunogenic and effective mRNA-LNP intranasal vaccine may also be uncovered as this burgeoning approach continues to be investigated and advanced in the field.

This preclinical study explored the immunogenicity and protective efficacy of intranasally administered mRNA-LNP vaccines using SARS-CoV-2 as a model pathogen. Overall, a two-dose primary intranasal vaccination regimen elicited systemic immune responses and resulted in lower SARS-CoV-2 infection levels and disease severity versus mock-vaccinated controls after viral challenge. In particular, two doses of mRNA-LNP2—a cationic LNP—elicited systemic immune responses at both low and high doses (5 and 25 μg). These titers were generally similar to the titers elicited by intramuscular controls at the respective suboptimal and protective doses (0.4 and 1.0 μg). Further, vaccination with mRNA-LNP2 reduced post-challenge viral titers in the lung and nasal turbinates relative to mRNA-LNP1 at the respective 5- and 25-μg dose levels, suggesting that an intranasally administered cationic LNP offers improved protection against SARS-CoV-2 compared to a noncationic formulation. Both intranasal vaccine formulations at the 25-μg level prevented severe lung pathology and reduced SARS-CoV-2 infection within the lungs. Together, these findings indicate that intranasal vaccination with an mRNA-LNP SARS-CoV-2 vaccine is protective and can induce systemic immune responses comparable to intramuscular controls, which have already been shown to be highly effective against COVID-19 (*21*, *22*, *45*). Notably, hamsters vaccinated with mRNA-LNP2 had greater levels of serum neutralizing antibodies against SARS-CoV-2 variant omicron B.1.1.529 compared with hamsters vaccinated with mRNA-LNP1. These encouraging data from sera would suggest that equivalent or improved results could be expected at mucosal sites; however, the role of antibody cross-neutralization at mucosal sites remains to be explored.

T and B cells at the site of infection are considered to be essential for effective protection of the mucosal tissues (*46*). Using immunohistochemistry to quantify lymphocytes present in the lung tissue on day 3 post-challenge intranasal high-dose mRNA-LNP2 increased the CD20^+^ B cell population in the lung tissue relative to mock vaccination, suggesting a quick B cell response. Overall, while the T cell population did not change in the high dose intranasal vaccination groups at 3 days after challenge, neither did it change in the high dose intramuscular vaccination control group. Multiple factors could contribute to this lack of change in the T cell population, ranging from the timing of tissue collection to the fixation method. In addition, CD3 is a pan T cell marker that does not distinguish between subtype (e.g., tissue resident, effector, memory, among others) nor does the assay examine antigen-specific T cells populations; these subpopulations could vary between intranasal vaccine groups as shown with the CD4^+^ T cell population.

The findings of this study should be considered alongside several limitations. First, efficacy assessments were performed in a preclinical animal model that is known to be highly susceptible to SARS-CoV-2 infection (*36*, *47*), which may not be immediately translatable to other animal models or human populations. In addition, S-specific mucosal IgA levels were not specifically measured due to both bronchoalveolar lavage and nasal wash procedures being terminal in hamsters; therefore, mucosal-specific antibody responses resulting from intranasal vaccination were not determined in this study. However, it would be expected that intranasal vaccination would elicit higher mucosal IgA immune responses in the respiratory tract than intramuscular vaccination (*39*, *41*). Our finding that intranasal vaccination with the higher dose level of mRNA-LNP2 induced comparable serum binding IgA titers to intramuscular controls remains encouraging. Notably, this study used higher dose levels for intranasal than intramuscular delivery, which is in line with recent evidence showing higher dose levels are required to successfully deliver sufficient product to target cells (such as epithelial cells) in the upper respiratory tract (*15*, *42*), likely because of physiological barriers inherent to the mucosa. The selected intranasal vaccine dose levels investigated in this study were exploratory and based on an unpublished pilot study in mice that focused on protein expression in the epithelial cells of the nasal passages after intranasal delivery; intramuscular dose levels were based on a previous study in hamsters (*35*) and used as controls for immunogenicity and protective efficacy. However, intranasal administration of mRNA-LNP vaccines is an emerging science, with substantial emphasis placed on improving intranasal mRNA-LNP delivery strategies to overcome inherent challenges unique to the mucosal environment of the nasal passages (*48*). Use of an intranasal spray or an aerosolization device could possibly benefit vaccine delivery by allowing for improved delivery throughout the respiratory tract.

Additional studies that further evaluate the potential advantages of intranasal vaccination in preclinical models other than an acute protection model should assist in translating these findings to clinical settings. Studies in ferrets (in addition to hamsters) can aid in examining the potential for intranasal vaccination to reduce transmission, while studies in mice and nonhuman primate models could enable investigating persistence of immune responses and the induction of local mucosal tissue-resident immunity and cellular immunity. However, the technically challenging nature of intranasal vaccination, coupled with the limited predictive power of preclinical intranasal vaccine findings for human populations, will need to be considered throughout the vaccine development. Intranasal vaccination as a booster regimen following primary parenteral vaccination schedules should also be evaluated, as an intranasal booster could build upon primary vaccination to supplement mucosal immunity and provide early, durable protection against infection.

In conclusion, we have demonstrated that intranasally administered mRNA-LNP vaccines delivered as a primary two-dose regimen are immunogenic and can protect naive hamsters from SARS-CoV-2 infection. Furthermore, we have shown that LNPs can be optimized and formulated for improved respiratory delivery with the addition of a cationic lipid, which increased immunogenicity. These encouraging findings may have broad implications for a progressive vaccination approach to supplement or complement current intramuscular approaches. Further investigations of intranasal mRNA-LNP vaccination alone or as a booster dose following an intramuscular primary series are thus warranted to address the burden of infectious respiratory disease worldwide. While an mRNA-based approach to intranasal vaccination may need to be further developed to reduce the effective dose level and increase suitability for human populations, our promising results show that mRNA-LNP vaccines have potential for effective administration via the intranasal route.

## METHODS

### Experimental design

Female Syrian golden hamsters (6 to 7 weeks old; Envigo) were intranasally vaccinated with a SARS-CoV-2 vaccine on a two-dose schedule with 3 weeks between doses (days 0 and 21; Fig. 1). Hamsters (*n* = 10 per vaccine group) were intranasally administered 40 μl (split between each naris) of SARS-CoV-2 vaccine (5 or 25 μg) formulated in two different LNP compositions; as a control, one group (*n* = 10) was administered with tris/sucrose buffer (mock vaccination) intranasally. An additional two groups (*n* = 10 per group) were intramuscularly vaccinated into the hind leg with the SARS-CoV-2 vaccine (0.4 or 1 μg), formulated with the preclinical version of the same LNP used in mRNA-1273. Serum samples for immunogenicity assessments were collected at 3 weeks after dose 1 (day 21) and 3 weeks after dose 2 (day 41).

At 21 days after dose 2 (day 42), all vaccinated hamsters were infected with 100 μl of (50 μl per naris) SARS-CoV-2 (2019-nCoV/USA-WA1/2020; Genbank: MN985325.1; courtesy of World Reference Center for Emerging Viruses and Arboviruses, University of Texas Medical Branch) at 10^5^ PFU. Through 14 days after viral challenge, hamsters were monitored daily for weight changes. At 3 and 14 days after infection, the lungs and nasal turbinates were collected from each vaccine group (*n* = 5 animals per time point). Before SARS-CoV-2 challenge, one animal each in the 5-μg and 25-μg mRNA-LNP2 groups died: One succumbed to territorial behavior, and the other cause of death was unknown. Animal experiments were carried out in compliance with approval from the Institutional Animal Care and Use Committee of the University of Texas Medical Branch.

### Preclinical mRNA and LNP production process

A sequence-optimized mRNA encoding the SARS-CoV-2 S protein with six proline mutations (*32*) was in vitro synthesized and purified as previously described (*27*). mRNA was LNP-encapsulated via nanoprecipitation by microfluidic mixing of ionizable, structural, helper, and polyethylene glycol lipids in acetate buffer (pH 5.0), followed by buffer exchange, concentration via tangential flow filtration, and filtration through a 0.8/0.2-μm membrane (*27*, *49*); an additional cationic lipid was added for mRNA-LNP2. The drug product was analytically characterized, and the products were evaluated as acceptable for in vivo use.

### S-2P-specific ELISA

MaxiSorp 96-well flat-bottom plates (Thermo Fisher Scientific) were coated with S-2P protein [1 μg/ml (for IgG) or 5 μg/ml (for IgA); GenScript] in 1× phosphate-buffered saline (PBS), corresponding to the spike protein of the Wuhan-Hu-1 virus stabilized with two proline mutations, and incubated at 4°C overnight. The plates were then washed four times with PBS + 0.05% Tween 20 and blocked with SuperBlock buffer in PBS (Thermo Fisher Scientific) for 1.5 hours at 37°C. After washing, fivefold serial dilutions of serum [assay diluent: PBS + 5% goat serum (Gibco) + 0.05% Tween 20) was added and incubated for 2 hours at 37°C (IgG) or overnight (IgA). Plates were washed, and bound antibodies were detected with horseradish peroxidase (HRP)–conjugated goat anti-hamster IgG antibodies (1:10,000; Abcam, AB7146) or HRP-conjugated rabbit anti-hamster IgA antibodies (1:5000; Brookwood Biomedical, sab3003) for 1 hour at 37°C. Plates were washed, and bound antibody detected with SureBlue TMB substrate (Kirkegaard & Perry Labs Inc.). After incubating at room temperature for 12 min, the 3,3,5,5-tetramethylbenzidine stop solution (Kirkegaard & Perry Labs Inc.) was added, and the absorbance was measured at 450 nM. GraphPad Prism (version 9.4.0) was used to determine titers using a four-parameter logistic curve fit for IgG or defined as the reciprocal dilution at approximately optical density (OD) for IgA with baseline defined as threefold above the OD of the blank.

### SARS-CoV-2 neutralization assay

Twofold dilutions of serum (heat inactivated, at an initial 1:10 dilution) were prepared in serum-free minimal essential media (MEM) and then incubated with SARS-CoV-2 [2019-nCoV/USA-WA01/2020 or B.1.1.529 (Omicron)] at a final concentration of 100 PFU at 37°C for 1 hour. Mixtures of virus-sera were then absorbed onto monolayers of Vero-E6 cells for 1 hour at 37°C in 96-well plates, then replaced with an overlay of MEM/methylcellulose/2% fetal bovine serum (FBS), and incubated for 2 or 3 days with 2019-nCoV/USA-WA01/2020 or B.1.1.529, respectively, at 37°C in humidified 5% CO_2_. Plaques were immunostained as described below for viral load analysis by plaque assay and then counted with the ImmunoSpot analyzer (Cellular Technology Limited); neutralization titers were determined at an end point of 60% plaque reduction.

### Analysis of viral load by plaque assay

The nasal turbinates and right lung were homogenized in Leibovitz L-15 medium (Thermo Fisher Scientific) supplemented with 10% FBS and 1× antibiotic-antimycotic by a TissueLyser II bead mill with 5-mm stainless steel beads (QIAGEN). After brief centrifugation, 10-fold serial dilutions of homogenates were prepared in serum-free MEM and then absorbed on 48-well plates of Vero-E6 monolayers for 1 hour at 37°C. The virus inoculum was removed, replaced with an overlay of MEM/methylcellulose/2% FBS, and incubated for 3 days. Plaques were then immunostained using a human monoclonal antibody cocktail specific for the SARS-CoV-2 S protein (clones DB_A03-09, 12; DB_B01-04, B07-10, 12; DB_C01-05, 07,09, 10; DB_D01, 02; DB_E01-04, 06, 07; and DB_F02-03; provided by Distributed Bio) and an anti-human IgG HRP-conjugated secondary antibody (catalog no. 5220-0456, Sera Care) and then counted to determine the virus load per gram of tissue.

### Analysis of viral load by qRT-PCR

Replicating viral RNA in the lung and nasal turbinates was determined via qRT-PCR measuring subgenomic SARS-CoV-2 E gene RNA using previously described primers, probe, and cycle conditions (*50*). In brief, RNA was extracted from homogenates using TRIzol LS (Thermo Fisher Scientific) and Direct-zol RNA Microprep kit (Zymo Research). Quantitative one-step real-time PCR was performed using extracted RNA (10 ng), TaqMan Fast Virus 1-step Master Mix (Thermo Fisher Scientific), primers, and a FAM-ZEN/Iowa Black FQ-labeled probe sequence (Integrated DNA Technologies) on the QuantStudio 6 system (Applied Biosystems). An Ultramer DNA oligonucleotide spanning the amplicon (Integrated DNA Technologies) was used for standard curve generation to calculate sgRNA copies per gram of tissue.

### Histopathology

The histological analysis of lung samples followed a standard protocol. In brief, the lower left lobe of the lung was fixed in 10% neutral buffered formalin, paraffin-embedded, sectioned (5 μm), and stained with hematoxylin and eosin. Sections were evaluated in a blinded manner by a board-certified veterinary pathologist under light microscopy with an Olympus BX51 microscope. Slides were scanned with a 20× (numerical aperture, 0.8) objective at a single layer with continuous stage movement scanning method, and images were captured using a Pannoramic 250 Flash III (3DHISTECH). Glass slides were examined, and microscopic diagnoses were graded independently on a five-level severity scale (grades 1 to 5: minimal, mild, moderate, marked, and severe) by two veterinary pathologists.

### Immunohistochemistry

Immunohistochemistry was performed on formalin-fixed paraffin embedded sections using the Leica Bond RX auto-stainer (Leica Microsystems). Specific details for each antibody used are provided in table S2. In brief, sections were baked for 1 hour before staining and dewaxed on the instrument. Antigen retrieval was then performed for 20 min at 95°C using Leica Epitope Retrieval Buffer 2 followed by treatment with Dako serum-free protein block (X090930-2, Agilent Dako) for 15 min to prevent nonspecific binding of the antibody. Tissue was then incubated with a given antibody for 30 min, and then antibody binding was detected using the Bond Polymer Refine Detection Kit (DS9800, Leica Microsystems) and bluing reagent (3802918, Leica Microsystems) to enhance the color. Images were taken at ×20 magnification using a Panoramic 250 Flash II scanner (3DHISTECH). Image analysis was performed using Halo software (Indica Labs). The percentage of positive cells was calculated as a ratio of positive cells for an antibody over the total cell population of the stained tissue. Macrophage marker IBA1 increases when macrophages are activated, which will result in an increased staining intensity of IBA1^+^ cells. Staining intensity of IBA1^+^ cells was quantified in in the Halo software through an *H* score, which designate the number of either weak, moderate, or strong signal cells and rate them on a scale to 300.

### Statistical modeling and hypothesis testing

Bayesian linear mixed model was used to model IgG, IgA, and neutralization titers, separately. A Bayesian model was chosen for its flexibility in model estimation when the data was censored (left at the limit of detection) and presented heterogeneous group variances. Because the Bayesian model was used for the ease of model fitting but not as a means to include prior information, we opt for noninformative prior in our analysis. For IgG, IgA, and neutralization titers (log_10_ titers), each dosing day was modeled separately with one main effect of composition and dose combination (six levels) and residual variance specific to each dose level (5, 25, 0.4, and 1 μg). Default priors in the brms R package was used, with noninformative flat priors used for all regression coefficients. Holm’s method was used to adjust *P* values for multiple comparisons. For viral loads (log_10_ transformed), ordinary linear regression was used with modeled data on day 3 only, as viral loads on day 14 were zero for all hamsters. Šidák’s method was used to adjust *P* values for multiple comparisons. All hypothesis testing was done two sided at alpha level of 0.05, except when noted otherwise. R version 4.1.2 was used for statistical modeling (*51*).

Kruskal-Wallis nonparametric test was implemented in hypothesis testing for image analysis using GraphPad’s Prism software. This form of analysis of variance (ANOVA) accounts for the small sample size in each experimental group and the small percentage of N-protein^+^ cells among animals in the vaccinated groups.
