# Supplementary material for: Intranasal mRNA-LNP vaccination protects hamsters from SARS-CoV-2 infection
Source: Sci Adv. 2023 Sep 22;9(38):eadh1655. doi: 10.1126/sciadv.adh1655 (PMC10516494; doi:10.1126/sciadv.adh1655)
Supplement: Supplementary file 1 — Figs. S1 to S3 Tables S1 to S8 [file sciadv.adh1655_sm.pdf]

Science Advances
AAAS

Supplementary Materials for

Intranasal mRNA-LNP vaccination protects hamsters from SARS-CoV-2 infection

Gabriela Baldeon Vaca *et al.*

Corresponding author: Kapil Bahl, kapil.bahl@modernatx.com;
Alexander Bukreyev, alexander.bukreyev@utmb.edu

Sci. Adv. **9**, eadh1655 (2023)
DOI: 10.1126/sciadv.adh1655

This PDF file includes:

Figs. S1 to S3
Tables S1 to S8

Fig. S1.

a

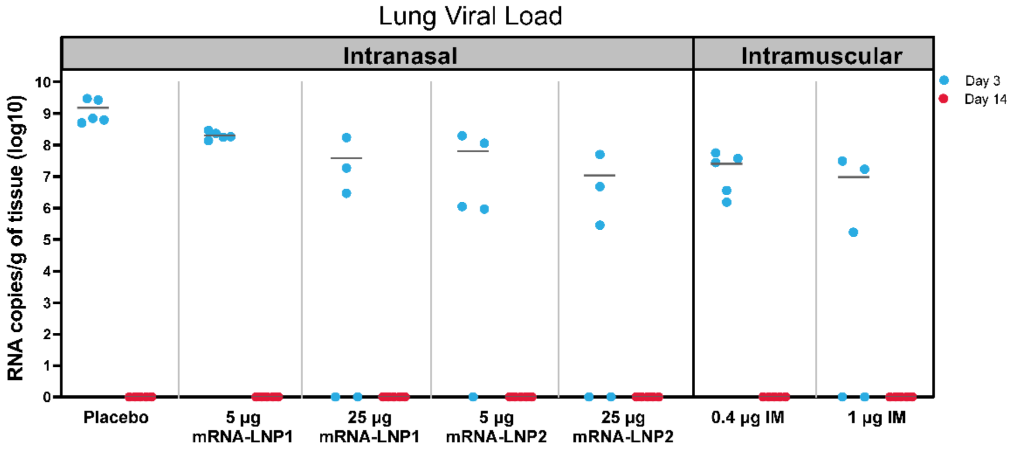

b

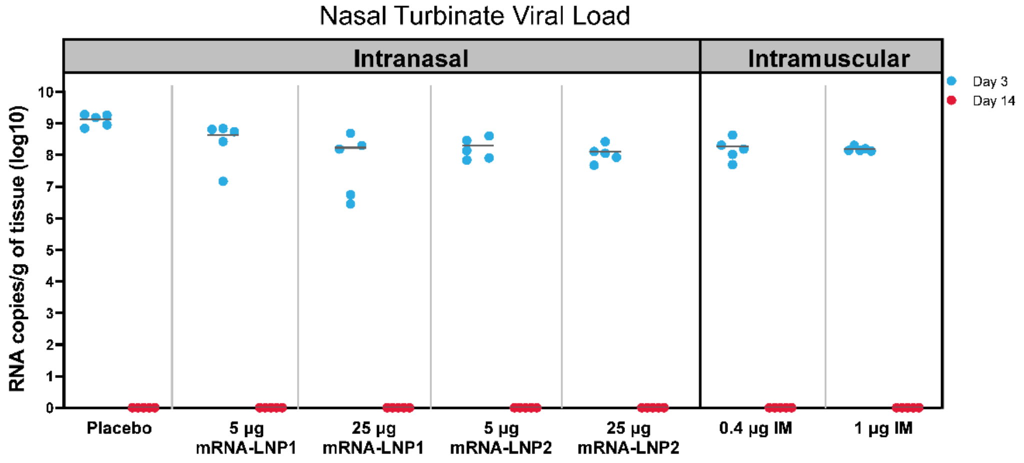

Fig. S1. Viral load as determined via qRT-PCR through 14 days after SARS-CoV-2 challenge in vaccinated hamsters. Viral load (sgRNA copies per gram of tissue) at 3 days and 14 days after SARS-CoV-2 challenge in (a) lungs and (b) nasal turbinates of vaccinated hamsters. Animal-level data are shown as dots (n = 5 animals per group), with the grey lines representing the geometric mean of each group. LLOD = 10^3 copies/g of tissue.

IM, intramuscular; IN, intranasal; LNP, lipid nanoparticle; mRNA, messenger RNA; qRT-PCR, quantitative reverse transcription polymerase chain reaction; SARS-CoV-2, severe acute respiratory syndrome coronavirus 2; sgRNA, subgenomic RNA.

Fig. S2.

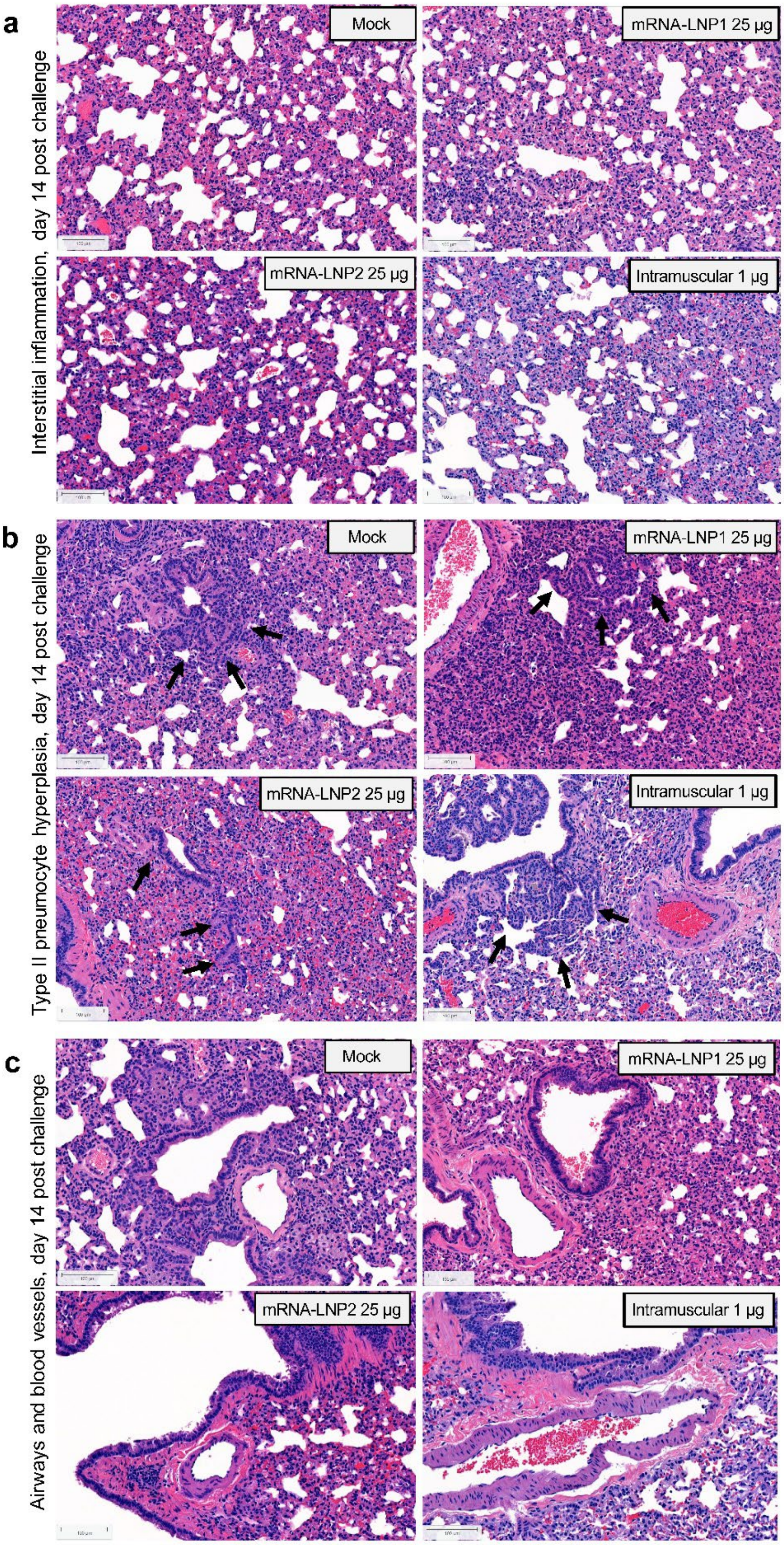
a
Interstitial inflammation, day 14 post challenge
Mock
mRNA-LNP1 25 μg
mRNA-LNP2 25 μg
Intramuscular 1 μg
b
Type II pneumocyte hyperplasia, day 14 post challenge
Mock
mRNA-LNP1 25 μg
mRNA-LNP2 25 μg
Intramuscular 1 μg
c
Airways and blood vessels, day 14 post challenge
Mock
mRNA-LNP1 25 μg
mRNA-LNP2 25 μg
Intramuscular 1 μg

Fig. S2. Pulmonary pathology characteristics at 14 days after SARS-CoV-2 challenge in vaccinated hamsters. Lung sections from hamsters at 14 days after SARS-CoV-2 challenge were stained with H&E. Representative images of (a) interstitial inflammation, (b) type II pneumocyte hyperplasia (arrows), or (c) airways and blood vessels are shown for hamsters intranasally administered 2 doses of tris/sucrose buffer (mock-vaccinated), mRNA-LNP1 (25 μg), mRNA-LNP2 (25 μg), or were intramuscularly vaccinated with 2 doses of vaccine (1.0 μg). Scale bars = 100 μm.

H&E, hematoxylin and eosin; IN, intranasal; SARS-CoV-2, severe acute respiratory syndrome coronavirus 2.

Fig. S3.

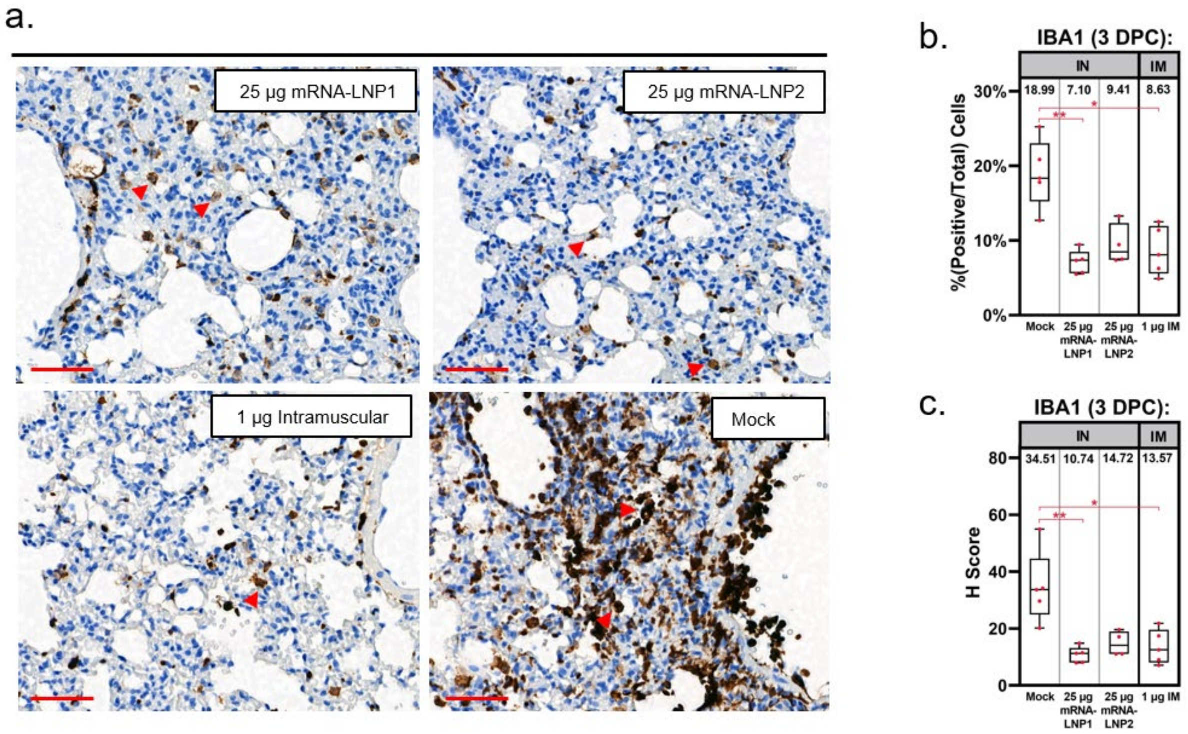

Fig. S3. Immunohistochemistry assessment of innate immune response via macrophage marker IBA1 at 3 days after SARS-CoV-2 challenge. Lungs sections from hamsters necropsied at 3 days post SARS-CoV-2 challenge were stained for macrophage marker IBA1. (a) Representational images of hamster lung with 2 doses of either tris/sucrose, 25 µg mRNA-LNP1, 25 µg mRNA-LNP2, or 1 µg IM composition. (b) Quantification of cells positive for IBA1 in each of the above represented groups. (c) Quantification of staining intensity of cells positive for IBA1 in each of the above represented groups. Scale bars represent 50 µm. Animal-level data are shown as dots (n = 4 to 5 animals per group), with boxes and horizontal bars denoting the IQR and median, respectively, and whiskers representing the maximum and minimum values. Means are stated above each boxplot. Kruskal-Wallis non-parametric test was implemented for statistical analysis to accommodate for small sample sizes per group. *$P < 0.05$; **$P < 0.01$; ***$P < 0.001$.

DPC, days post challenge; IBA1, ionized calcium-binding adapter molecule 1; IM, intramuscular; IN, intranasal; LNP, lipid nanoparticle; mRNA, messenger RNA; SARS-CoV-2, severe acute respiratory syndrome coronavirus 2.

Table S1. Statistical comparisons of ancestral S-specific serum binding IgG antibody titers after vaccination

Comparison	Estimated Fold-change (95% CI)	Adjusted P-value
Dose 1 (Day 21)		
25-µg mRNA-LNP1 over 5-µg mRNA-LNP1	68.53 (25.63-185.69)	<1e-6
25-µg mRNA-LNP2 over 5-µg mRNA-LNP1	67.68 (25.04-182.43)	<1e-6
5-µg mRNA-LNP2 over 5-µg mRNA-LNP1	50.61 (22.1-117.24)	<1e-6
25-µg mRNA-LNP1 over 0.4-µg IM	3.11 (0.86-11.06)	0.9619
25-µg mRNA-LNP1 over 1-µg IM	1.63 (0.61-4.38)	0.8426
5-µg mRNA-LNP1 over 0.4-µg IM	0.05 (0.02-0.14)	<1e-6
5-µg mRNA-LNP1 over 1-µg IM	0.02 (0.01-0.05)	<1e-6
25-µg mRNA-LNP2 over 25-µg mRNA-LNP1	0.99 (0.31-3.14)	0.4892
25-µg mRNA-LNP2 over 5-µg mRNA-LNP2	1.34 (0.5-3.71)	0.7183
25-µg mRNA-LNP2 over 0.4-µg IM	3.07 (0.9-11.02)	0.9635
25-µg mRNA-LNP2 over 1-µg IM	1.61 (0.6-4.4)	0.8312
5-µg mRNA-LNP2 over 25-µg mRNA-LNP1	0.74 (0.27-2.03)	0.2668
5-µg mRNA-LNP2 over 0.4-µg IM	2.3 (0.75-6.89)	0.9359
5-µg mRNA-LNP2 over 1-µg IM	1.2 (0.54-2.69)	0.6883
1-µg IM over 0.4-µg IM	1.91 (0.63-5.83)	0.8853
Dose 2 (Day 41)		
25-µg mRNA-LNP1 over 5-µg mRNA-LNP1	3.64 (1.54-8.55)	0.0024
25-µg mRNA-LNP2 over 25-µg mRNA-LNP1	3.68 (1.78-7.55)	6e-04
25-µg mRNA-LNP2 over 5-µg mRNA-LNP1	13.38 (5.58-31.97)	<1e-6
25-µg mRNA-LNP2 over 5-µg mRNA-LNP2	2.7 (1.1-6.61)	0.0156

5-µg mRNA-LNP2 over 5-µg mRNA-LNP1	4.95 (1.8-13.8)	0.0023
25-µg mRNA-LNP1 over 0.4-µg IM	0.61 (0.27-1.34)	0.1004
25-µg mRNA-LNP1 over 1-µg IM	0.37 (0.17-0.86)	0.0138
5-µg mRNA-LNP1 over 0.4-µg IM	0.17 (0.06-0.43)	2e-04
5-µg mRNA-LNP1 over 1-µg IM	0.1 (0.04-0.27)	2e-04
25-µg mRNA-LNP2 over 0.4-µg IM	2.24 (0.99-4.93)	0.9742
25-µg mRNA-LNP2 over 1-µg IM	1.38 (0.61-3.2)	0.7796
5-µg mRNA-LNP2 over 25-µg mRNA-LNP1	1.36 (0.56-3.32)	0.7592
5-µg mRNA-LNP2 over 0.4-µg IM	0.83 (0.32-2.27)	0.3406
5-µg mRNA-LNP2 over 1-µg IM	0.51 (0.19-1.38)	0.0894
1-µg IM over 0.4-µg IM	1.63 (0.63-3.98)	0.8621

CI, confidence interval; IgG, immunoglobulin G; IM, intramuscular; LNP, lipid nanoparticle; mRNA, messenger RNA.

Table S2. Statistical comparisons of ancestral S-specific serum binding IgA antibody titers after vaccination

Comparison	Estimated Fold-change (95% CI)	Adjusted P-value
Dose 1 (Day 21)		
25-µg mRNA-LNP2 over 25-µg mRNA-LNP1	83.81 (38.04-224.25)	<1e-6
25-µg mRNA-LNP2 over 5-µg mRNA-LNP2	32.7 (3.92-716.22)	0.0035
25-µg mRNA-LNP2 over 0.4-µg IM	2.84 (1.18-6.73)	0.0104
25-µg mRNA-LNP1 over 0.4-µg IM	0.03 (0.01-0.09)	<1e-6
25-µg mRNA-LNP1 over 1-µg IM	0.02 (0.01-0.05)	<1e-6
25-µg mRNA-LNP2 over 1-µg IM	1.7 (0.72-3.93)	0.8993
5-µg mRNA-LNP2 over 25-µg mRNA-LNP1	2.56 (0.12-24.18)	0.8043
5-µg mRNA-LNP2 over 0.4-µg IM	0.09 (<1e-6-0.8)	0.0171
5-µg mRNA-LNP2 over 1-µg IM	0.05 (<1e-6-0.44)	0.0066
1-µg IM over 0.4-µg IM	1.67 (0.67-4.14)	0.8746
Dose 2 (Day 41)		
25-µg mRNA-LNP1 over 5-µg mRNA-LNP1	3.74 (1.37-10.19)	0.0051
25-µg mRNA-LNP2 over 25-µg mRNA-LNP1	5.77 (2.57-12.95)	<1e-6
25-µg mRNA-LNP2 over 5-µg mRNA-LNP1	21.57 (7.93-60.5)	<1e-6
25-µg mRNA-LNP2 over 5-µg mRNA-LNP2	4.05 (1.37-11.73)	0.0066
5-µg mRNA-LNP2 over 5-µg mRNA-LNP1	5.33 (1.55-18.18)	0.006
25-µg mRNA-LNP1 over 0.4-µg IM	0.21 (0.08-0.52)	0.0025
25-µg mRNA-LNP1 over 1-µg IM	0.13 (0.05-0.33)	<1e-6
5-µg mRNA-LNP1 over 0.4-µg IM	0.06 (0.02-0.18)	<1e-6
5-µg mRNA-LNP1 over 1-µg IM	0.03 (0.01-0.1)	<1e-6
25-µg mRNA-LNP2 over 0.4-µg IM	1.21 (0.47-3.09)	0.6658
25-µg mRNA-LNP2 over 1-µg IM	0.73 (0.28-1.88)	0.2411
5-µg mRNA-LNP2 over 25-µg mRNA-LNP1	1.43 (0.51-4.12)	0.7531

5-µg mRNA-LNP2 over 0.4-µg IM	0.3 (0.09-0.95)	0.0219
5-µg mRNA-LNP2 over 1-µg IM	0.18 (0.06-0.57)	0.0044
1-µg IM over 0.4-µg IM	1.67 (0.58-4.79)	0.8428

CI, confidence interval; IgA, immunoglobulin A; IM, intramuscular; LNP, lipid nanoparticle; mRNA, messenger RNA.

Table S3. Statistical comparisons of serum neutralizing antibody titers against ancestral SARS-CoV-2 after vaccination

Comparison	Estimated Fold-change (95% CI)	Adjusted P-value
Dose 1 (Day 21)		
25-µg mRNA-LNP2 over 25-µg mRNA-LNP1	38.84 (8.7-287.37)	<1e-6
25-µg mRNA-LNP2 over 5-µg mRNA-LNP2	17.46 (2.21-275.24)	0.0044
25-µg mRNA-LNP2 over 0.4-µg IM	8.09 (1.09-96.32)	0.0205
25-µg mRNA-LNP1 over 0.4-µg IM	0.21 (0.02-2.64)	0.0801
25-µg mRNA-LNP1 over 1-µg IM	0.06 (0.01-0.21)	<1e-6
25-µg mRNA-LNP2 over 1-µg IM	2.16 (0.63-7.5)	0.8996
5-µg mRNA-LNP2 over 25-µg mRNA-LNP1	2.22 (0.12-24.4)	0.7781
5-µg mRNA-LNP2 over 0.4-µg IM	0.46 (0.02-7.72)	0.275
5-µg mRNA-LNP2 over 1-µg IM	0.12 (0.01-0.77)	0.0149
1-µg IM over 0.4-µg IM	3.74 (0.6-39.29)	0.9309
Dose 2 (Day 41)		
25-µg mRNA-LNP1 over 5-µg mRNA-LNP1	5.07 (1.53-18.22)	0.0054
25-µg mRNA-LNP2 over 25-µg mRNA-LNP1	5.7 (2.28-14.07)	0.0008
25-µg mRNA-LNP2 over 5-µg mRNA-LNP1	28.89 (8.5-111.28)	<1e-6
25-µg mRNA-LNP2 over 5-µg mRNA-LNP2	5.08 (1.46-18.06)	0.0059
5-µg mRNA-LNP2 over 5-µg mRNA-LNP1	5.69 (1.32-27.13)	0.0119
25-µg mRNA-LNP1 over 0.4-µg IM	0.36 (0.14-0.91)	0.0173
25-µg mRNA-LNP1 over 1-µg IM	0.17 (0.06-0.45)	0.0004
5-µg mRNA-LNP1 over 0.4-µg IM	0.07 (0.02-.024)	0.0002
5-µg mRNA-LNP1 over 1-µg IM	0.03 (0.01-0.12)	<1e-6
25-µg mRNA-LNP2 over 0.4-µg IM	2.06 (0.83-5.26)	0.94
25-µg mRNA-LNP2 over 1-µg IM	0.96 (0.33-2.65)	0.4737
5-µg mRNA-LNP2 over 25-µg mRNA-LNP1	1.12	0.5776

	(0.32-3.86)	
5-µg mRNA-LNP2 over 0.4-µg IM	0.41 (0.12-1.43)	0.0758
5-µg mRNA-LNP2 over 1-µg IM	0.19 (0.05-0.69)	0.0065
1-µg IM over 0.4-µg IM	2.15 (0.77-6.24)	0.9333

CI, confidence interval, IM, intramuscular; LNP, lipid nanoparticle; mRNA, messenger RNA.

Table S4. Statistical comparisons of viral load by plaque assay[a] in the lungs of vaccinated hamsters at 3 days after SARS-CoV-2 challenge

Comparison	Fold-change (standard error)	*t*-ratio	P-value
0.4-µg IM over 1-µg IM	2.32 (1.23)	1.88	0.783
0.4-µg IM over 25-µg mRNA-LNP1	1.15 (1.17)	0.98	1
0.4-µg IM over 5-µg mRNA-LNP1	-1.91 (1.11)	-1.72	0.881
0.4-µg IM over 25-µg mRNA-LNP2	2.77 (1.05)	2.64	0.245
0.4-µg IM over 5-µg mRNA-LNP2	0.99 (0.98)	1.01	1
0.4-µg IM over mock	-2.60 (0.90)	-2.88	0.148
1-µg IM over 25-µg mRNA-LNP1	0.01 (1.23)	0.01	1
1-µg IM over 5-µg mRNA-LNP1	-3.05 (1.17)	-2.60	0.266
1-µg IM over 25-µg mRNA-LNP2	1.63 (1.11)	1.46	0.97
1-µg IM over 5-µg mRNA-LNP2	-0.15 (1.05)	-0.15	1
1-µg IM over mock	-3.73 (0.98)	-3.82	0.014
25-µg mRNA-LNP1 over 5-µg mRNA-LNP1	-1.88 (1.23)	-1.53	0.955
25-µg mRNA-LNP1 over 25-µg mRNA-LNP2	2.79 (1.17)	2.39	0.401
25-µg mRNA-LNP1 over 5-µg mRNA-LNP2	1.02 (1.11)	0.91	1
25-µg mRNA-LNP1 over mock	-2.57 (1.05)	-2.45	0.354
5-µg mRNA-LNP1 over 25-µg mRNA-LNP2	5.85 (1.23)	4.76	0.001
5-µg mRNA-LNP1 over 5-µg mRNA-LNP2	4.07 (1.17)	3.48	0.035
5-µg mRNA-LNP1 over mock	0.49 (1.11)	0.44	1
25-µg mRNA-LNP2 over 5-µg mRNA-LNP2	-0.60 (1.23)	-0.49	1
25-µg mRNA-LNP2 over mock	-4.18 (1.17)	-3.57	0.027
5-µg mRNA-LNP2 over mock	-2.40 (1.23)	-1.96	0.731

[a]Viral loads (log_{10} transformed) were assessed by ordinary linear regression; only modeled data at Day 3 after challenge was evaluated since viral loads on Day 14 were zero for all hamsters. A degree of freedom of 28 was used.

IM, intramuscular; LNP, lipid nanoparticle; mRNA, messenger RNA.

Table S5. Statistical comparisons of viral load by plaque assay in the nasal turbinates of vaccinated hamsters at 3 days after SARS-CoV-2 challenge

Comparison	Fold-change (standard error)	*t*-ratio	P-value
0.4-µg IM over 1-µg IM	3.09 (0.87)	3.56	0.028
0.4-µg IM over 25-µg mRNA-LNP1	0.55 (0.83)	0.67	1
0.4-µg IM over 5-µg mRNA-LNP1	-0.97 (0.78)	-1.24	0.995
0.4-µg IM over 25-µg mRNA-LNP2	2.59 (0.74)	3.52	0.031
0.4-µg IM over 5-µg mRNA-LNP2	1.30 (0.69)	1.89	0.779
0.4-µg IM over mock	-2.02 (0.64)	-3.18	0.073
1-µg IM over 25-µg mRNA-LNP1	-1.53 (0.87)	-1.76	0.86
1-µg IM over 5-µg mRNA-LNP1	-3.05 (0.83)	-3.69	0.02
1-µg IM over 25-µg mRNA-LNP2	0.52 (0.78)	0.66	1
1-µg IM over 5-µg mRNA-LNP2	-0.78 (0.74)	-1.05	0.999
1-µg IM over mock	-4.09 (0.69)	-5.95	0
25-µg mRNA-LNP1 over 5-µg mRNA-LNP1	-0.51 (0.87)	-0.59	1
25-µg mRNA-LNP1 over 25-µg mRNA-LNP2	3.06 (0.83)	3.70	0.019
25-µg mRNA-LNP1 over 5-µg mRNA-LNP2	1.76 (0.78)	2.25	0.5
25-µg mRNA-LNP1 over mock	-1.56 (0.74)	-2.11	0.607
5-µg mRNA-LNP1 over 25-µg mRNA-LNP2	4.58 (0.87)	5.29	0
5-µg mRNA-LNP1 over 5-µg mRNA-LNP2	3.28 (0.83)	3.98	0.009
5-µg mRNA-LNP1 over mock	-0.03 (0.78)	-0.04	1
25-µg mRNA-LNP2 over 5-µg mRNA-LNP2	-0.28 (0.87)	-0.33	1
25-µg mRNA-LNP2 over mock	-3.60 (0.83)	-4.36	0.003
5-µg mRNA-LNP2 over mock	-2.31 (0.87)	-2.66	0.236

[a]Viral loads (log_{10} transformed) were assessed by ordinary linear regression; only modeled data at Day 3 after challenge was evaluated since viral loads on Day 14 were zero for all hamsters. A degree of freedom of 28 was used.

IM, intramuscular; LNP, lipid nanoparticle; mRNA, messenger RNA.

Table S6. Major pulmonary histopathological findings and severity scores after SARS-CoV-2 challenge by vaccine group

Day 3 (After Challenge)							
		Intranasal				Intramuscular	
	Mock (n = 5)	mRNA-LNP1 5 µg (n = 5)	mRNA-LNP1 25 µg (n = 5)	mRNA-LNP2 5 µg (n = 5)	mRNA-LNP2 25 µg (n = 5)	0.4 µg (n = 5)	1 µg (n = 5)
Interstitial inflammation, n							
Group total	5	5	5	5	5	5	5
2 (mild)	2	4	2	2	1	5	2
3 (moderate)	3	1	3	3	4	0	3
Bronchial/bronchiolar inflammation, n							
Group total	4	5	1	3	1	3	4
1 (minimal)	2	1	1	1	0	2	3
2 (mild)	0	3	0	2	1	1	1
3 (moderate)	2	1	0	0	0	0	0
Vascular inflammation, n							
Group total	4	3	2	3	2	4	1
1 (minimal)	0	1	1	1	1	3	0
2 (mild)	2	2	1	1	0	1	1
3 (moderate)	2	0	0	1	1	0	0

Day 14 (After Challenge)							
		Intranasal				Intramuscular	
	Mock (n = 5)	mRNA-LNP1 5 µg (n = 5)	mRNA-LNP1 25 µg (n = 5)	mRNA-LNP2 5 µg (n = 4)	mRNA-LNP2 25 µg (n = 4)	0.4 µg (n = 5)	1 µg (n = 5)
Interstitial inflammation, n							
Group total	5	5	5	4	4	5	5
2 (mild)	0	5	4	0	4	4	3
3 (moderate)	5	0	1	4	0	1	2
Type II pneumocyte hyperplasia, n							
Group total	5	4	5	4	2	1	5
1 (minimal)	0	3	3	1	2	0	0
2 (mild)	4	1	1	2	0	0	3
3 (moderate)	1	0	1	1	0	1	2
Bronchial/bronchiolar inflammation, n							
Group total	4	3	2	3	1	1	3
1 (minimal)	2	3	2	1	1	1	3
2 (mild)	2	0	0	2	0	0	0
Vascular inflammation, n							
Group total	1	4	4	4	1	5	3
1 (minimal)	1	4	4	4	1	5	3

IM, intramuscular; LNP, lipid nanoparticle; mRNA, messenger RNA; SARS-CoV-2, severe acute respiratory syndrome coronavirus 2.

Table S7. Summary of hamsters positive for SARS-CoV-2 N-protein at 3 days after challenge

Vaccine Group	Administration Route	Ratio of N-protein Positive Hamsters to Total Population (n/N)
Mock	IN	5/5
mRNA-LNP1 5 µg	IN	5/5
mRNA-LNP1 25 µg	IN	2/5
mRNA-LNP2 5 µg	IN	4/5
mRNA-LNP2 25 µg	IN	4/5
Intramuscular 0.4 µg	IM	3/5
Intramuscular 1 µg	IM	2/5

IN, intranasal; IM, intramuscular; LNP, lipid nanoparticle; mRNA, messenger RNA; N protein, nucleocapsid protein; SARS-CoV-2, severe acute respiratory syndrome coronavirus 2.

Table S8. Antibody Experimental Details

Antibody	Target	Clone	Company (Cat No.)	Dilution (Vol:Vol)	Antigen Retrieval Buffer
SARS-CoV-2 N-Protein	Covid infected cells	N/A	GeneTex (GTX135357)	(1:4K)	1
IBA1	Macrophages	EPR16588	Abcam (ab178846)	(1:10K)	1
CD3	T cells (general)	SP162	Abcam (ab135372)	(1:300)	2
CD20	B cells	SP32	Abcam (ab64088)	(1:100)	2
CD4	Helper T Cells	D7D2Z	CST (77699S)	(1:100)	1

*(Vol:Vol) is the ratio of volume of antibody to solution it is diluted in; **N/A: Not applicable.
